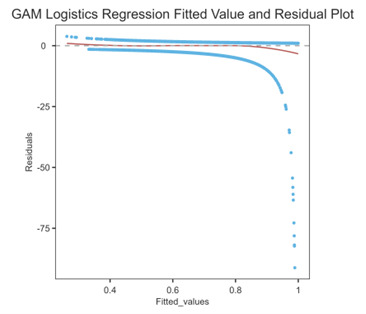# Association between estimated pulse wave velocity and acute kidney injury in critically ill sepsis patients: A MIMIC-IV database analysis

**DOI:** 10.1097/MD.0000000000049272

**Published:** 2026-06-12

**Authors:** Yulai Wu, Qingqing Lin, Guowei Li, Xiaoyan Liu, Jing Nie, Lihua Wang, Fengmin Ge, Zhiyi Li

**Affiliations:** a Department of Anesthesiology, Affiliated Hospital of Guangdong Medical University, Zhanjiang, China; b Faculty of Chinese Medicine, Macau University of Science and Technology, Macau, China.

**Keywords:** acute kidney injury, MIMIC-IV database, pulse wave analysis, sepsis

## Abstract

The incidence of acute kidney injury (AKI) in patients undergoing coronary revascularization is significantly associated with estimated pulse wave velocity (ePWV). While many studies have confirmed the effect of ePWV on AKI, its significance in the progression of AKI among critically ill patients with sepsis remains unclear. This study utilized a retrospective cohort design using data from the Medical Information Mart for Intensive Care-IV database, which included adult intensive care unit patients with sepsis who were admitted for at least 48 hours. ePWV was derived from the patient’s age and blood pressure. Participants were divided into 2 groups depending on whether they had AKI. The relationship between ePWV and AKI risk in sepsis patients was thoroughly assessed through univariate analysis, multivariate logistic regression, threshold effect analysis, and restricted cubic spline modeling. Analysis of the Medical Information Mart for Intensive Care-IV database showed that 66.1% of 18,351 sepsis patients developed AKI. In model 3, ePWV was significantly associated with AKI risk (odds ratio 1.02, 95% confidence interval: 1.01–1.03; *P* = .027). Restricted cubic spline analysis revealed a nonlinear dose-response relationship between ePWV and AKI risk (*P* for nonlinearity = .004). Threshold analysis identified an inflection point around 12.275 m/s, although estimates in the higher ePWV range should be interpreted cautiously. Subgroup analyses confirmed that this association remained stable and consistent across most of the subgroups. ePWV independently predicted AKI risk in patients with sepsis. This straightforward, noninvasive metric can facilitate early risk stratification and intervention.

## 1. Introduction

Recently, noninvasive ePWV has been proposed as a practical surrogate marker of arterial stiffness based on readily available clinical parameters including age and blood pressure.^[1]^ Previous studies have demonstrated that ePWV is closely correlated with carotid-femoral pulse wave velocity, with reported differences of approximately 0.3%, supporting its validity as a reliable alternative measurement.^[2]^ In addition, ePWV has shown prognostic value for postoperative acute kidney injury (AKI) and mortality in surgical populations such as patients undergoing colorectal surgery.^[3]^

However, although there is increasing recognition that arterial stiffness contributes to microvascular dysfunction and organ damage, the role of ePWV in the development of AKI among critically ill patients with sepsis remains insufficiently investigated. Sepsis is characterized by severe hemodynamic instability, systemic inflammation, and endothelial dysfunction, all of which may exacerbate arterial stiffness and predispose patients to renal injury.^[4]^ Whether ePWV can serve as an early noninvasive indicator of AKI risk in high-risk populations has not been systematically studied.

Therefore, this study aimed to examine the association between ePWV and AKI incidence in patients with acute sepsis. By focusing on a large intensive care cohort, this study sought to extend the application of clinically derived ePWV to the critical care context and evaluate its potential utility as a rapid and accessible tool for risk stratification of AKI in patients with sepsis within the intensive care setting.

## 2. Materials and methods

### 2.1. Data source

This study used data from the Medical Information Mart for Intensive Care (MIMIC)-IV database, which contains clinical records for over 70,000 ICU admissions at the Beth Israel Deaconess Medical Center in Boston, from 2008 to 2022. The Institutional Review Board at the Massachusetts Institute of Technology approved the study protocol, ensuring that the dataset was thoroughly anonymized. Yulai Wu successfully completed the certified data access training program (Authorization Code: 67727247) and was responsible for overseeing the data extraction.To ensure strict confidentiality, all patient information was anonymized prior to data processing and analysis.The research methodologies complied with internationally recognized ethical standards for human participant research.

### 2.2. Study population

**Figure 1.**
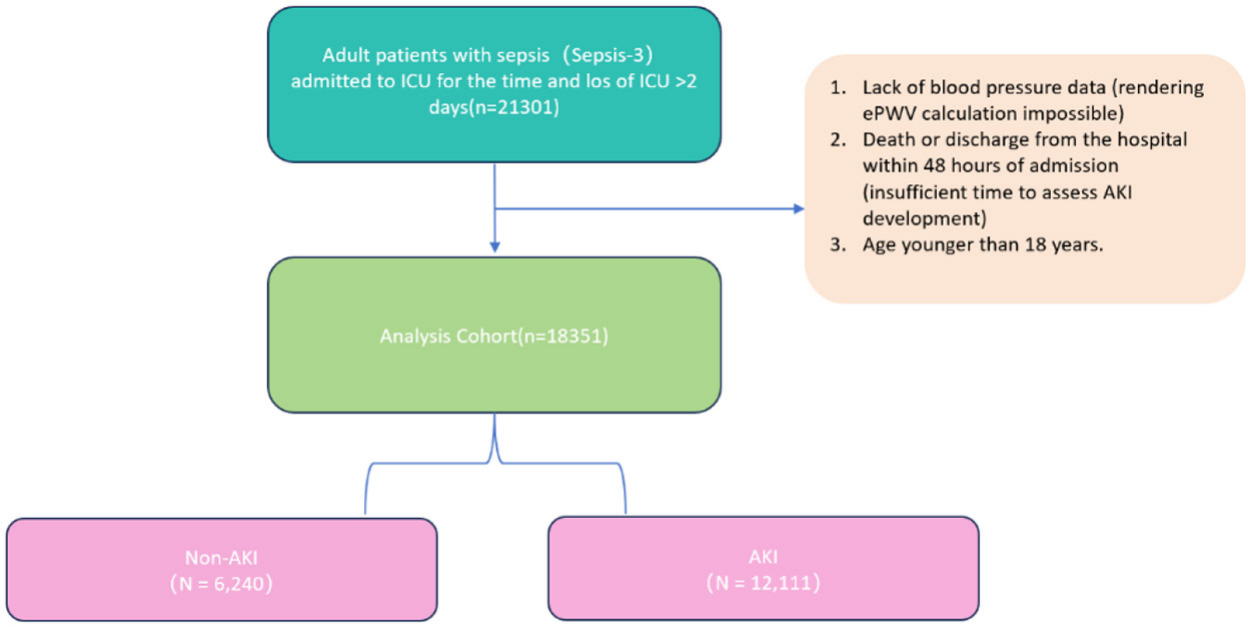
The flowchart of patients’ selection. AKI = acute kidney injury, ePWV = estimated pulse wave velocity, ICU = intensive care unit.

We enrolled adult patients (≥18 years) meeting the Sepsis-3.0 criteria, requiring an ICU stay of at least 48 hours. Sepsis-3.0 necessitates a SOFA score of ≥2 points along with confirmed or suspected infection.^[5]^ AKI identification was primarily based on the KDIGO (Kidney Disease: Improving Global Outcomes) Clinical Practice Guidelines, which define it based on serum creatinine levels and urine output.^[6]^ Patients without blood pressure data or those discharged or deceased within 48 hours of admission were excluded from the study. According to the KDIGO Clinical Practice Guidelines, AKI is defined and staged based on serum creatinine levels and urine output criteria, encompassing KDIGO stages 1, 2, and 3. We chose to include all stages of AKI, as even mild renal impairment (stage 1) has been associated with adverse outcomes in critically ill patients with sepsis.^[7]^ The study included 18,351 patients who were divided into 2 groups according to the occurrence of AKI (Fig. 1).

### 2.3. Data collection and processing

Patient demographic and laboratory test data were systematically collected using Navicat Premium 16, with a focus on vital signs, such as red blood cell, WBC, and platelet counts, along with kidney function indicators, such as blood urea nitrogen (BUN) and serum creatinine. Multiple imputations were performed using the multiple imputation by chained equations algorithm for variables with missing rates below 30%. To assess the impact of data imputation on statistical inference, a sensitivity analysis was conducted using complete-case data. The results are presented in Table S2, Supplemental Digital Content 1 and Figure S2, Supplemental Digital Content 2. The initial blood pressure value recorded upon ICU admission was used for blood pressure assessment. For those with multiple records within 2 hour, the average of the first 3 was used. Mean arterial pressure (MAP) was determined using the formula MAP = DBP + 0.4 × (SBP − DBP).^[8]^ The ePWV was determined using the following formula^[2,9]^:


$$\begin{array}{c} ePWV=9.587-0.402\times age+4.560\times 10^{-3}\\ \times \;age^{2}-2.621\times 10^{-5}\times age^{2}\times MAP\\ +3.176\times 10^{-3}\times age\times MAP-1.832\times 10^{-2}\times MAP \end{array}$$

### 2.4. Statistical analysis

All statistical analyses were performed using the R software (version 4.3.3; R Foundation for Statistical Computing). Statistical significance was defined as a 2-sided *P*-value < .05, unless otherwise specified.

Data on baseline characteristics and laboratory parameters were extracted from the first 24 hours of ICU admission. Continuous variables with missingness <30% were imputed using multiple imputation by chained equations, generating 5 complete datasets. Logistic regression was used for categorical variables, and linear regression was used for continuous variables. Variables with >30% missing data were excluded.

Continuous variables following a normal distribution are presented as mean ± standard deviation and compared using Student *t* test (or ANOVA for variables with more than 2 groups). Non-normally distributed continuous variables are expressed as median (interquartile range, IQR), and group comparisons are performed using the Mann–Whitney *U* test (or Kruskal–Wallis test for multi-group comparisons). Categorical variables are summarized as frequencies with percentages, and between-group differences are assessed using Pearson’s chi-squared test (or Fisher’s exact test if expected cell counts were less than 5).

A univariate logistic regression model was used to identify potential covariates (Table 2). Variables with a *P* value < .05 in the univariate analysis were considered statistically significant and included in the subsequent multivariable model.

The primary outcome was the incidence of AKI. The independent association between ePWV and AKI was assessed using multivariable logistic regression with a hierarchical adjustment strategy across the 3 models to progressively control for confounding factors:

Model 1 (crude): unadjusted.
Model 2 (partially adjusted): adjusted for demographic variables of race and sex.
Model 3 (fully adjusted): additionally adjusted for all significant covariates identified in the univariate analysis, including age, SpO_2_, platelets, WBC, albumin, anion gap, calcium, chloride, glucose, potassium, sodium, lactate, INR, prothrombin time, serum creatinine, comorbidities (hypertension [HTN], cancer, diabetes, heart failure, myocardial infarction [MI], coronary heart disease (CHD), chronic obstructive pulmonary disease (COPD), liver cirrhosis, pneumonia [PNA], cerebral infarction, and chronic bronchitis [CB]), interventions (vasopressin use), and illness severity (Sequential Organ Failure Assessment score, SOFA). Table 3 presents the results.

**Figure 2.**
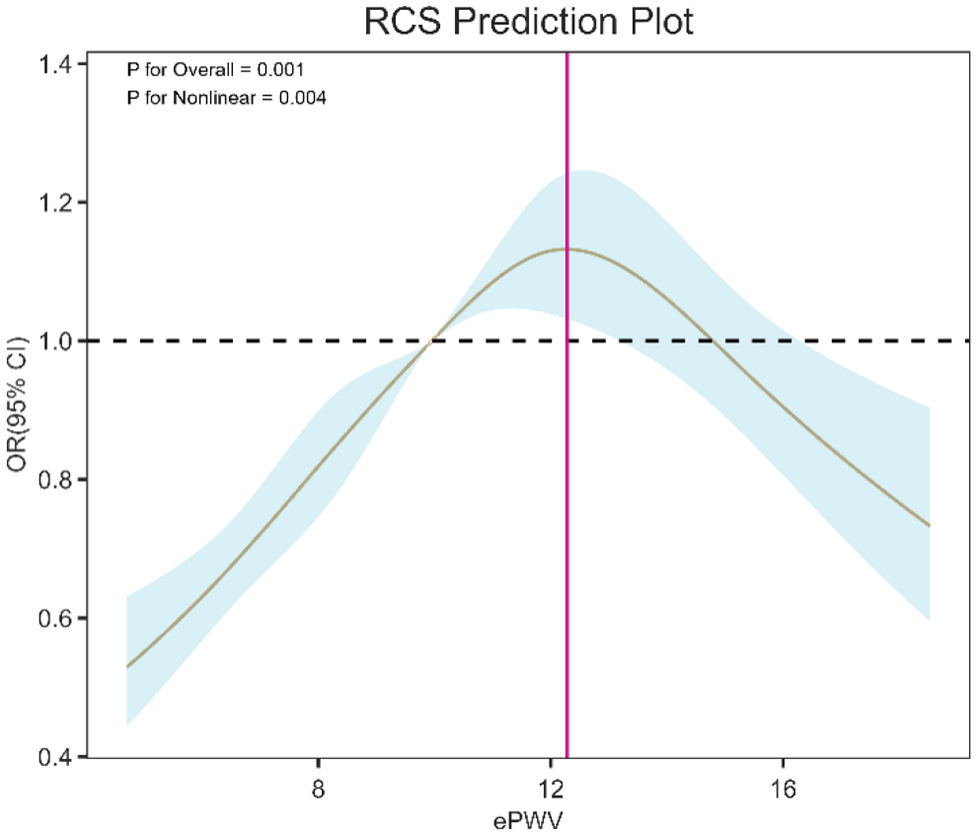
RCS curve for the ePWV odds ratio and AKI. The RCS curve is set with 5 nodes, and the RCS knot positions (m/s) are (9.95, 8.27, 6.18, 15.40, 11.92). The RCS curve shows the relationship between ePWV and AKI in sepsis patients in the MIMIC database. RCS demonstrates the correlation between ePWV and AKI in the fully adjusted logistic regression model. The nonlinear *P* value indicates whether there is a nonlinear correlation between ePWV and the outcome, and *P* < .05 indicates a nonlinear correlation. AKI = acute kidney injury, ePWV = estimated pulse wave velocity, MIMIC = Medical Information Mart for Intensive Care, RCS = restricted cubic spline

To assess potential nonlinearity in the dose-response relationship, restricted cubic spline (RCS) analysis was conducted within the fully adjusted logistic regression framework (model 3), using 5 knots placed at prespecified percentiles. The overall statistical significance of the nonlinear components was tested using the likelihood ratio test. The RCS curve is shown in Figure 2.

To validate the robustness of the nonlinear pattern further, a Generalized Additive Model (GAM) with penalized regression splines was fitted as an independent smoothing method. The results of GAM validation are shown in Figure S1, Supplemental Digital Content 3.

In light of the significant nonlinearity detected, a 2-piecewise logistic regression model was employed to precisely quantify the threshold effect (Table 4). The inflection point was determined by maximizing the likelihood of the model. Model fit was compared with a simple linear model using a likelihood ratio test.

**Figure 3.**
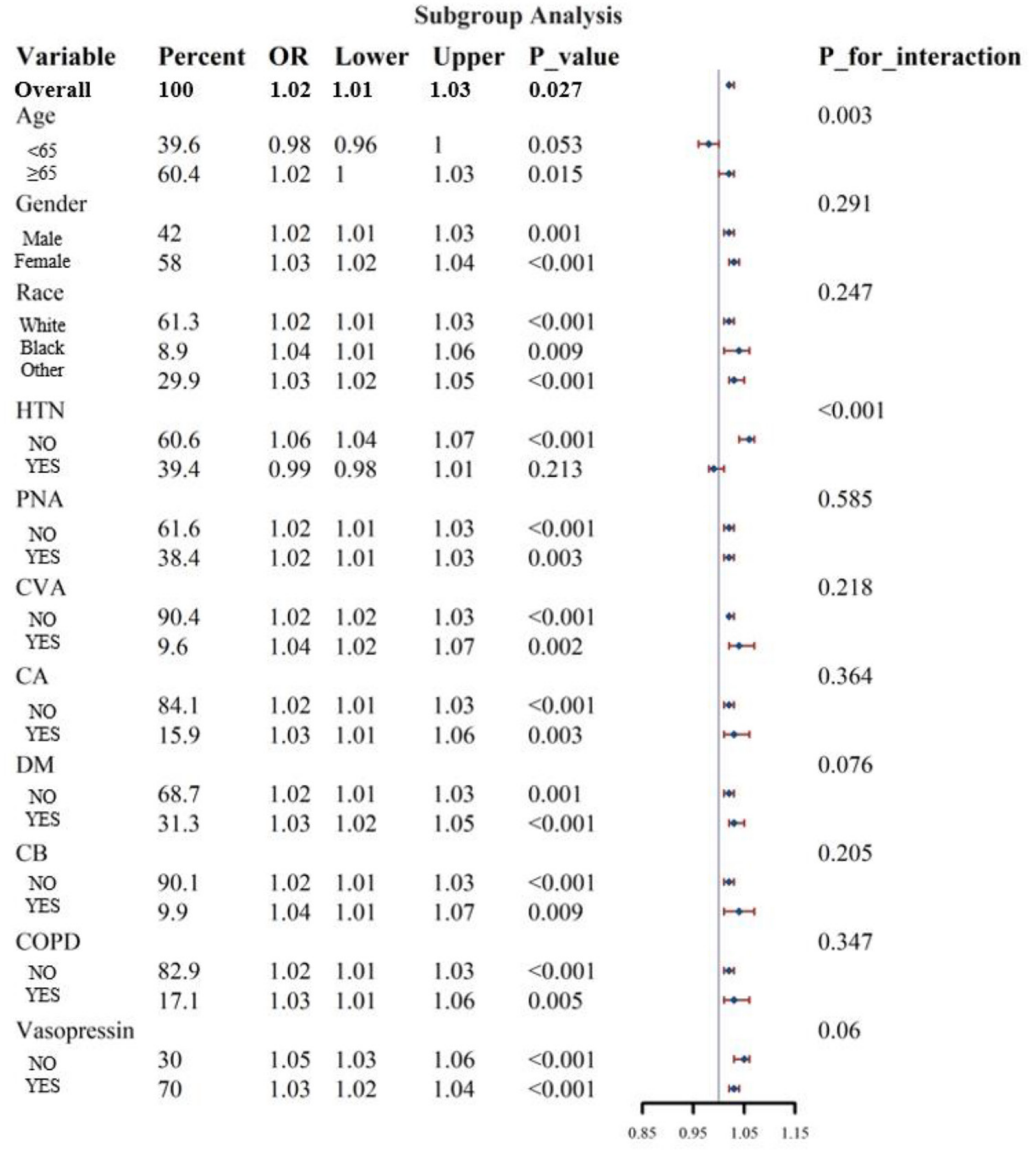
Subgroup analysis. The analysis presents odds ratios and 95% confidence intervals associated with a 1 m/s increase in ePWV. The bold horizontal line at the top represents the overall effect, adjusted for all covariates in model 3 (adjusted OR = 1.02, 95% CI: 1.01–1.03, *P* = .027). Subgroup analyses were performed according to age, sex, race, use of vasoactive drugs, and key comorbidities. *P* values for interaction tests for effect modification across subgroups are shown. CI = confidence interval, ePWV = estimated pulse wave velocity OR = odds ratio.

Stratified analyses are performed to examine the consistency of the ePWV-AKI association across predefined subgroups (Fig. 3) including age, sex, race, and key comorbidities. The interaction terms are tested using multiplicative models.

## 3. Results

### 3.1. Baseline characteristics

This study included 18,351 patients (Fig. 1). Table 1 shows the baseline characteristics of the patients in the non-AKI (n = 6240) and AKI (n = 12,111) groups. In the non-AKI group, the median ePWV was 9.61 with an interquartile range of 4.25, whereas in the AKI group, it was 10.13 with an interquartile range of 4.03. Patients in the AKI group were older, predominantly male, and had elevated levels of SBP, diastolic blood pressure (DBP), MAP, SPO_2_, platelet count, red blood cell, albumin, anion gap, glucose, potassium, SCR, and BUN compared to those in the non-AKI group. The AKI group exhibited a higher prevalence of comorbidities including HTN, diabetes mellitus (DM), heart failure (HF), cancer (CA), CHD, PNA, and COPD. The 28-day ICU mortality rate was significantly higher in the AKI group (10.18%) than in the non-AKI group (4.81%).

**Table 1:** Baseline characteristics of septic patients stratified by the occurrence of AKI.

Categories	Overall (N = 18,351)	Non-AKI (N = 6240)	AKI (N = 12,111)	*P*
Age (yr)	67.00 (21.00)	65.00 (23.00)	68.00 (21.00)	<.001
Gender:male	10,534.00 (57.40%)	3447.00 (55.24%)	7087.00 (58.52%)	<.001
Race				.284
White	11,725.00 (63.89%)	3938.00 (63.11%)	7787.00 (64.30%)	
Black	1348.00 (7.35%)	468.00 (7.50%)	880.00 (7.27%)	
Other	5278.00 (28.76%)	1834.00 (29.39%)	3444.00 (28.44%)	
SBP (mm Hg)	119.00 (32.00)	119.00 (30.00)	118.00 (33.00)	.037
DBP (mm Hg)	66.00 (22.00)	67.00 (21.00)	65.00 (21.00)	<.001
MAP (mm Hg)	87.00 (22.80)	88.00 (22.20)	86.40 (23.40)	<.001
SpO_2_ (%)	99.00 (4.00)	98.00 (4.00)	99.00 (4.00)	<.001
Laboratory tests				
Platelets (K/μL)	182.00 (119.00)	179.00 (120.00)	184.00 (118.00)	<.001
WBC (10^9^/L)	3.50 (1.01)	3.51 (0.98)	3.49 (1.03)	.159
RBC (10^12^/L)	11.20 (7.40)	10.40 (7.30)	11.60 (7.50)	<.001
Albumin (g/dL)	3.02 (0.02)	3.02 (0.08)	3.03 (0.02)	<.001
Aniongap (mEq/L)	13.00 (5.00)	13.00 (4.00)	13.00 (5.00)	<0.001
Calcium (mg/dL)	8.30 (0.80)	8.29 (0.90)	8.30 (0.80)	.344
Chloride (mEq/L)	105.00 (8.00)	105.00 (8.00)	105.00 (8.00)	.062
Glucose (mg/dL)	125.00 (52.00)	120.00 (48.00)	127.00 (53.00)	<.001
Potassium (mEq/L)	4.10 (0.80)	4.00 (0.70)	4.10 (0.70)	<.001
Sodium (mmol/L)	139.00 (5.00)	139.00 (5.00)	139.00 (5.00)	.015
Lactate (mmol/L)	2.17 (1.00)	2.17 (0.77)	2.10 (1.10)	<.001
INR	1.30 (0.40)	1.30 (0.35)	1.30 (0.30)	<.001
PT (s)	14.40 (3.30)	14.20 (3.25)	14.50 (3.60)	<.001
SCR (mg/dL)	0.80 (0.40)	0.80 (0.40)	0.90 (0.40)	<.001
BUN (mg/dL)	17.00 (11.00)	15.00 (10.00)	17.00 (12.00)	<.001
Commorbidities				
HTN	8284.00 (45.14%)	2584.00 (41.41%)	5700.00 (47.06%)	<.001
CA	2946.00 (16.05%)	1002.00 (16.06%)	1944.00 (16.05%)	.991
DM	4691.00 (25.56%)	1342.00 (21.51%)	3349.00 (27.65%)	<.001
HF	4258.00 (23.20%)	1074.00 (17.21%)	3184.00 (26.29%)	<.001
MI	954.00 (5.20%)	227.00 (3.64%)	727.00 (6.00%)	<.001
CHD	6486.00 (35.34%)	1774.00 (28.43%)	4712.00 (38.91%)	<.001
COPD	2632.00 (14.34%)	830.00 (13.30%)	1802.00 (14.88%)	.004
LC	1210.00 (6.59%)	422.00 (6.76%)	788.00 (6.51%)	.507
PNA	6782.00 (38.36%)	3011.00 (33.46%)	3771.00 (43.44%)	<.001
CVA	1693.00 (9.58%)	933.00 (10.37%)	760.00 (8.76%)	<.001
CB	1754.00 (9.92%)	717.00 (7.97%)	1037.00 (11.95%)	<.001
28-D mortality	1533.00 (8.35%)	300.00 (4.81%)	1233.00 (10.18%)	<.001
Vasopressin				<.001
No	5605.00 (30.54%)	3500.00 (36.19%)	2105.00 (24.25%)	
Yes	12,746.00 (69.46%)	6171.00 (63.81%)	6575.00 (75.75%)	
SOFA	6.00 (5.00)	5.00 (4.00)	7.00 (5.00)	<.001
ePWV (m/s)	9.95 (4.13)	9.61 (4.25)	10.13 (4.03)	<.001

BUN = blood urea nitrogen, CA = cancer, CB = chronic bronchitis, CHD = coronary heart disease, COPD = chronic obstructive pulmonary disease, CVA = cerebrovascular accident, DBP = diastolic blood pressure, DM = diabetes mellitus, ePWV = estimated pulse wave velocity, HF = heart failure, HTN = hypertension, INR = international normalized ratio, LC = liver cirrhosis, MAP = mean arterial pressure, MI = myocardial infarction, PNA = pneumonia, PT = prothrombin time, RBC = red blood cell, SBP = systolic blood pressure, SCR = creatinine, SOFA = Sequential Organ Failure Assessment, WBC = white blood cell.

### 3.2. Univariate logistic regression

Univariate logistic regression analysis (Table 2) was initially conducted to identify the factors potentially influencing the occurrence of AKI in patients with sepsis. The analysis provided odds ratio (OR), confidence interval (CI), and *P* values to quantify the strength and statistical significance of these associations. The findings indicated that multiple variables were significantly associated with a heightened risk of AKI (*P* < .05). Among demographic factors, age ≥ 65 years (OR = 1.39, 95% CI: 1.39–1.48, *P* < .001) and male sex (OR = 1.14, 95% CI: 1.07–1.22, *P* < .001) were associated with a higher risk. Comorbidities such as HF (OR = 1.72, 95% CI: 1.59–1.85, *P* < .001), MI (OR = 1.69, 95% CI: 1.46–1.97, *P* < .001), CHD (OR = 1.60, 95% CI: 1.50–1.71, *P* < .001), DM (OR = 1.40, 95% CI: 1.30–1.50, *P* < .001), HTN (OR = 1.26, 95% CI: 1.18–1.34, *P* < .001), PNA (OR = 1.53, 95% CI: 1.44–1.62, *P* < .001), CB (OR = 1.57, 95% CI: 1.42–1.73, *P* < .001), and COPD (OR = 1.14, 95% CI: 1.04–1.25, *P* = .003) were linked to an increased risk of AKI, with HF showing the strongest correlation. In addition, we found several laboratory indicators related to an increased risk of AKI, including elevated potassium (OR = 1.36, 95% CI: 1.29–1.42, *P* < .001), SCR (OR = 1.37, 95% CI: 1.32–1.43, *P* < .001), lactate (OR = 1.10, 95% CI: 1.07–1.13, *P* < .001), SOFA (OR = 1.21, 95% CI: 1.20–1.22, *P* < .001), and anion gap (OR = 1.04, 95% CI: 1.03–1.05, *P* < .001). Conversely, albumin demonstrated a negative correlation with risk (OR = 0.80, 95% CI: 0.74–0.86, *P* < .001). Among vital signs, SpO_2_ exhibited a mild association with risk (OR = 1.02, 95% CI: 1.01–1.03, *P* < .001). The primary indicator, ePWV, demonstrated a significant positive correlation (OR = 1.05, 95% CI: 1.04–1.06, *P* < .001), establishing a basis for future multivariate analyses to verify its independent predictive value. This preliminary analysis identified various potential factors influencing SA-AKI and established a basis for the selection of variables in the multivariate models.

**Table 2:** Univariate logistic regression for AKI binary outcomes.

Variables	*P*	OR (95% CI)
Age, ≥65, yr	**<.001**	1.39 (1.31–1.48)
Gender, male	**<.001**	1.14 (1.07–1.22)
Race		
White		1.00 (Reference)
Black	.410	0.95 (0.85–1.07)
Other	.140	0.95 (0.89–1.02)
SpO_2_ (%)	**<.001**	1.02 (1.01–1.03)
HTN	**<.001**	1.26 (1.18–1.34)
DM	**<.001**	1.40 (1.30–1.50)
HF	**<.001**	1.72 (1.59–1.85)
MI	**<.001**	1.69 (1.46–1.97)
CHD	**<.001**	1.60 (1.50–1.71)
COPD	**.003**	1.14 (1.04–1.25)
PNA	**<.001**	1.53 (1.44–1.62)
CVA	**.003**	0.83 (0.75–0.92)
CB	**<.001**	1.57 (1.42–1.73)
Platelets (K/uL)	**<.001**	1.00 (1.00–1.00)
Albumin (g/dL)	**<.001**	0.80 (0.74–0.86)
RBC (K/uL)	0.160	0.98 (0.93–1.01)
WBC (K/μL)	**<.001**	1.02 (1.01–1.02)
Anion gap (mEq/L)	**<.001**	1.04 (1.03–1.05)
Glucose (mg/dL)	**<.001**	1.00 (1.00–1.00)
Potassium (mEq/L)	**<.001**	1.36 (1.29–1.42)
Sodium (mmol/L)	**.017**	1.01 (1.00–1.01)
Lactate (mmol/L)	**<.001**	1.10 (1.07–1.13)
INR	**<.001**	1.22 (1.16–1.30)
PT (s)	**<.001**	1.02 (1.01–1.02)
SCR (mg/dL)	**<.001**	1.37 (1.32–1.43)
BUN (mg/dL)	**<.001**	1.02 (1.02–1.02)
SOFA	**<.001**	1.21 (1.20–1.22)
ePWV (m/s)	**<.001**	1.05 (1.04–1.06)

Bold values indicate statistical significance, *P* < .05.

BUN = blood urea nitrogen, CB = chronic bronchitis, CHD = coronary heart disease, COPD = chronic obstructive pulmonary disease, CVA = cerebrovascular accident, DM = diabetes mellitus, ePWV = estimated pulse wave velocity, HF = heart failure, HTN = hypertension, INR = international normalized ratio, MI = myocardial infarction, PNA = pneumonia, PT = prothrombin time, RBC = red blood cell, SCR = creatinine, SOFA = Sequential Organ Failure Assessment, WBC = white blood cell.

### 3.3. Association between ePWV and AKI risk: multivariate regression and nonlinear analysis

The independent association between ePWV and AKI risk was assessed using multivariate logistic regression with hierarchical adjustment (Table 3). Variables for adjustment were selected based on clinical relevance and statistical significance (*P* < .05) in the univariate logistic regression analysis (Table 2). Three models were constructed to progressively control for confounding variables: model 1 (crude), model 2 (adjusted for race and sex), and model 3 (fully adjusted for all significant clinical and laboratory covariates: age, SpO_2_, platelets, WBC, albumin, anion gap, calcium, chloride, glucose, potassium, sodium, lactate, INR, prothrombin time, SCR, and comorbidities including HTN, CA, DM, HF, MI, CHD, COPD, liver cirrhosis, PNA, cerebrovascular accident, CB, vasopressin use, and SOFA score).

**Table 3:** Multivariate regression.

Variables	Model 1		Model 2		Model 3	
	OR (95% CI)	*P*	OR (95% CI)	*P*	OR (95% CI)	*P*
ePWV	1.05 (1.04–1.06)	<.001	1.05 (1.04–1.06)	<.001	1.02 (1.01–1.03)	.027
ePWV						
Q1	Ref		Ref		Ref	
Q2	1.31 (1.20–1.43)	<.001	1.30 (1.20–1.42)	<.001	1.12 (1.02–1.23)	<.005
Q3	1.54 (1.41–1.68)	<.001	1.54 (1.41–1.68)	<.001	1.24 (1.13–1.37)	<.001
Q4	1.47 (1.35–1.60)	<.001	1.48 (1.36–1.62)	<.001	1.15 (1.04–1.26)	<.004
*P* for trend	<.001		<.001		<.001	

Q1: (4.27–8.08); Q2: (8.08–9.95); Q3: (9.95–12.20); Q4: (12.21–19.44).

Model 1: crude model.

Model 2: adjusted race and gender.

Model3: adjusted SPO_2_, platelets, WBC, RBC, albumin, anion gap, calcium, chloride, glucose, potassium, sodium, lactate, INR, PT, SCR, HTN, CA, DM, HF, MI, CHD, COPD, LC, PNA,CVA, CB, vasopressin, and SOFA.

CA = cancer, CI = confidence interval, CHD = coronary heart disease, COPD = chronic obstructive pulmonary disease, CVA = cerebrovascular accident, DM = diabetes mellitus ePWV = estimated pulse wave velocity, HF = heart failure, HTN = hypertension, INR = international normalized ratio, LC = liver cirrhosis, MI = myocardial infarction, OR = odds ratio, PNA = pneumonia, PT = prothrombin time, RBC = red blood cell, Ref = reference, SCR = creatinine, SOFA = Sequential Organ Failure Assessment, SPO_2_ = oxygen saturation, WBC = white blood cell.

In the fully adjusted model 3, when ePWV was examined as a linear continuous variable, each 1 m/s increase was associated with a 2% higher odds of AKI (OR 1.02, 95% CI: 1.01–1.03, *P* = .027). To test for a potential nonlinear dose-response relationship, we primarily employed RCS analysis with 5 knots located at prespecified percentiles. The RCS curve revealed a significant inverted U-shaped association (*P* for nonlinearity = .004; Fig. 2).

To verify the robustness of this nonlinear pattern rigorously, we employed a GAM with penalized regression splines. The GAM-derived smooth curve consistently reproduced the same inverted U-shaped relationship (see Fig. S1, Supplemental Digital Content 3), and the smooth term for the ePWV was statistically significant (edf = 2.858, *P* < .001), providing independent confirmation. To exclude the potential influence of extreme values, we performed a sensitivity analysis by restricting the RCS to patients with ePWV < 15 m/s, and the nonlinear relationship remained statistically significant (Fig. S3, Supplemental Digital Content 4).

Given the established nonlinearity, a 2-piecewise logistic regression model was fitted to quantify the threshold effect precisely (Table 4). The model identified an inflection point at 12.275 m/s. Below this threshold, each 1 m/s increase in ePWV was strongly associated with a 5% higher odds of AKI (OR 1.05, 95% CI: 1.03–1.07; *P* < .001). Conversely, at or above 12.275 m/s, the association direction reversed, with each 1 m/s increase linked to an 8% lower odds of AKI (OR 0.92, 95% CI: 0.89–0.97, *P* < .001). A likelihood ratio test showed that this 2-piecewise model provided a significantly better fit than the simple linear model (*P* < .001), suggesting a threshold-dependent relationship between ePWV and AKI risk.

**Table 4:** Threshold effect analysis.

Outcome	OR (95% CI)	*P*
AKI		
ePWV per 1 m/s change		
Model 1 line effect	1.02 (1.01–1.03)	.030
Model 2 threshold		
Inflection point (m/s)		
<12.275	1.05 (1.03–1.07)	<.001
≥12.275	0.92 (0.89–0.97)	<.001
*P* for likelihood		<.001

Threshold effect analysis revealed a nonlinear (inflection point) association between ePWV and the risk of AKI. When ePWV was below the inflection point value of 12.275 m/s, each 1 m/s increase in ePWV was significantly associated with a higher risk of AKI (OR = 1.05, *P* < .001). However, when ePWV exceeded this threshold, it showed a negative correlation with AKI risk (OR = 0.92, *P* < .001), indicating that further increases in ePWV were associated with a reduced risk. The likelihood ratio test yielded *P* < .001, confirming that the segmented model was significantly superior to the linear model, thereby underscoring the presence of a complex threshold effect in the relationship between ePWV and AKI risk.

AKI = acute kidney injury, ePWV = estimated pulse wave velocity, OR = odds ratio.

### 3.4. Subgroup analysis

Subgroup analyses were conducted to evaluate the consistency of the association between ePWV and AKI incidence among septic patients categorized by age, sex, race, HTN, PNA, CA, DM, CB, COPD, cerebrovascular accident, and vasopressor use. As illustrated in Figure 3, these analyses demonstrated that the effect estimates for most subgroups were aligned with the overall study outcomes.

A significant positive correlation between elevated ePWV and AKI incidence in patients with sepsis was observed across different subgroups. These subgroups included patients aged ≥65 years, both female and male, individuals from all racial backgrounds, those with no history of HTN, those with and without PNA, CA, DM, CB, COPD, and those with or without vasopressor use. The absence of significant differences in patients aged <65 years or those with HTN may be attributed to imbalanced sample sizes, which could have resulted in insufficient statistical power.

Notably, the relationship between ePWV and AKI risk was particularly evident in the age and HTN subgroups (*P* for interaction < .05). This suggests that the effect of ePWV on the risk of AKI may be modulated by these variables.

## 4. Discussion

An elevated ePWV was significantly associated with the incidence of AKI in critically ill patients with sepsis and served as an independent prognostic factor. Increased ePWV remained a significant predictor of heightened AKI risk even after accounting for potential confounding variables. Further analyses identified a nonlinear relationship between ePWV and AKI incidence.

In patients with sepsis, early identification of AKI is crucial for reducing mortality rates. Nonetheless, accurate, convenient, and noninvasive biomarkers for AKI detection remain lacking. Clinically, the most frequently used quantifiable markers include SCR, BUN, urinary casts, and urine-concentrating ability. SCR is not highly effective for early AKI detection because of its delayed response to renal function changes and reliance on factors such as age, sex, and muscle mass.^[10]^ Arterial stiffness, associated with an elevated risk of cardiovascular mortality, may serve as a potential mechanism connecting kidney dysfunction to cardiovascular events, especially in highly vascularized kidneys. Systemic inflammation can impair kidney function, leading to alterations in the blood vessels and increased arterial stiffness.^[11]^ Recent research has indicated a link between increased aortic stiffness, brachial-ankle pulse wave velocity (baPWV), and systemic inflammation.^[12]^ Additionally, renal ischemia and tubular injury can result in fluid and electrolyte imbalances such as fluid retention and HTN, which may influence pulse wave velocity.^[13]^ Neurohormonal activation, characterized by increased sympathetic nervous system and renin-angiotensin-aldosterone system activity, is pivotal to this process.^[14]^ Consequently, neurogenic increases in vascular tone may serve as a direct pathological link between AKI and elevated arterial stiffness.^[15]^

Recent research has indicated that ePWV is independently associated with the development of diabetic kidney disease. Correlation analyses revealed a linear relationship between ePWV and urinary albumin-to-creatinine ratio, indicating its potential as an early screening tool for diabetic kidney disease. In cardiovascular medicine, elevated ePWV is significantly associated with higher all-cause mortality (HR 1.44–4.09) and cardiovascular mortality (HR 2.34–5.76), with risks notably increasing when ePWV exceeds 11.15 m/s.^[16]^ The study demonstrated that ePWV independently heightened the risk of AKI in critically ill patients with sepsis and showed a significant nonlinear association with AKI incidence.

An increased ePWV directly indicates reduced systemic vascular elasticity and acts as a marker of arterial stiffness. In sepsis, the systemic inflammatory response induces endothelial dysfunction and the consequent vascular stiffening associated with increased ePWV further impairs renal perfusion. Research shows that septic patients with increased ePWV face a notably higher risk of 28-day mortality (HR = 1.53, 95% CI: 1.36–1.71).^[17]^ Sepsis can harm renal tubular epithelial cells via pro-inflammatory cytokines such as interleukin-8 (IL-8) and tumor necrosis factor-alpha (TNF-α), in addition to oxidative stress.^[18]^ Elevated ePWV may amplify the detrimental effects of inflammatory mediators on renal function, as arterial stiffness exacerbates these adverse outcomes.^[19]^ Ferroptosis, a lipid peroxidation-dependent cell death, is more prone to activation in patients with sepsis and increased ePWV.^[20,21]^ Research has shown that the association between PWV and CKD is significantly affected by BMI and diabetes. In patients with sepsis, hyperglycemia and metabolic disturbances may exacerbate tubular injury owing to microcirculatory dysfunction associated with ePWV.^[22]^ Increased ePWV was independently correlated with both in-hospital and ICU mortality in ischemic stroke patients and showed a linear dose-response relationship with 28-day mortality in SA-AKI patients, without evidence of nonlinearity (*P* = .602). This association is particularly pronounced in patients with AKI who also suffer from congestive heart failure, suggesting that dysfunction within the cardiac-kidney axis may serve as a mechanism through which ePWV exacerbates AKI.^[23]^ In conclusion, increased ePWV contributes to the onset and aggravation of sepsis-related AKI through various mechanisms, including increased vascular stiffness, amplified inflammatory responses, and metabolic imbalances, all of which are characterized by complex multisystem interactions.

A RCS model adjusted for covariates demonstrated a significant nonlinear relationship between ePWV levels and AKI risk (*P* for nonlinear = .004). Threshold effect analysis revealed an inflection point of approximately 12.275 m/s (Table 4). A 1-unit rise in ePWV below this threshold correlated with a progressive increase in AKI risk.However, as the ePWV exceeded 12.275 m/s, the risk of AKI began to decrease, approaching a relative risk of 1.0, suggesting that further increases in ePWV did not significantly impact AKI risk.These results collectively illustrate an inverted U-shaped exposure–response relationship, where the risk of AKI peaks at a moderate ePWV level of approximately 12.275 m/s and is comparatively lower at both lower and substantially higher ePWV levels.In addition, the nonlinear relationship revealed by the RCS exhibited good robustness. This pattern was independently confirmed by a GAM, where the fitted smooth curve (see Fig. S1, Supplemental Digital Content 3) consistently demonstrated an inverted U-shaped association and the smooth term s(epwv) was highly significant (*P* < .001).

Subgroup analysis showed no notable differences in patients with HTN; however, a significant interaction existed between ePWV and AKI risk in this group (*P* for interaction < .001). This interaction may be attributed to the fact that HTN often leads to atherosclerosis and endothelial dysfunction. Because ePWV serves as an effective marker of vascular elasticity, its impact is more readily detectable in the absence of preexisting vascular damage. Conversely, in patients with HTN, the vasculature may already be compromised owing to chronic injury, late-stage compensatory mechanisms, or remodeling processes, potentially obscuring or even reversing the effects of ePWV.^[24]^ Furthermore, an interaction effect was observed in the age subgroups. In patients aged 65 years and older, ePWV was a significant risk factor for AKI (OR = 1.17), whereas in those younger than 65 years, it was not statistically significant (OR = 0.81). This phenomenon may be attributed to the fact that older populations typically demonstrate more pronounced vascular stiffening, with ePWV generally increasing with age.^[25]^ It is possible that the baseline level of vascular stiffness in these populations had already reached a threshold, thereby diminishing the ability of further increases in ePWV to effectively discriminate the risk of AKI. Previous research has indicated that ePWV is generally elevated in the elderly population, which may account for the decreased predictive sensitivity for AKI in these groups.

In patients with sepsis exhibiting an elevated ePWV, it is imperative for clinicians to intensify hemodynamic monitoring to prevent renal damage resulting from insufficient renal perfusion. ePWV is a useful tool for the risk stratification and prognostic assessment of AKI. Management strategies should be tailored to individual patient characteristics, including comorbidities and ePWV levels, while simultaneously prioritizing the preservation of both arterial elasticity and renal function. Nonetheless, further research is required to substantiate the impact of interventions aimed at mitigating the ePWV-associated AKI risk in the context of sepsis.

Notably, elevated ePWV may exacerbate the risk of AKI in patients with sepsis through multiple mechanisms. Arteriosclerosis has been demonstrated to directly impair the autoregulation of renal microcirculation, leading to insufficient renal perfusion.^[26]^ Concurrently, the inflammatory response activates endothelial cells, resulting in the release of vasoactive substances (e.g., endothelin-1) and pro-inflammatory factors (e.g., IL-6, TNF-α).^[27]^ This disrupts the glomerular filtration barrier and promotes oxidative stress. Furthermore, pulsatile blood flow caused by increased arterial stiffness may exacerbate tubular damage through the ferroptosis pathway in renal tubular epithelial cells (dependent on iron ions and lipid peroxidation).^[28]^ These mechanisms indirectly illustrate the potential value of vascular stiffness indicators for preventing sepsis-related AKI.

Although this study yielded significant findings, several limitations should be considered. The study exclusively utilized data from the MIMIC-IV database, and its retrospective design requires further validation of the causal link between elevated ePWV and AKI risk in patients with sepsis through prospective cohort studies. We note that for ePWV > 15 m/s, the sample size is relatively small, which may reduce statistical power and make estimates in this extreme range less reliable. Therefore, interpretations for this high ePWV range should be made cautiously, while the association in the middle range remains robust. This study focused exclusively on ICU-admitted patients with sepsis, potentially influencing the evaluation of hemodynamic status. Future studies should explore the association between ePWV and AKI in patients with sepsis beyond the ICU environment. Blood pressure measurements in this study may have been influenced by vasoactive drugs, which could have introduced bias into the baseline blood pressure values. This study also has limitations regarding the early diagnosis of AKI. Although it integrated creatinine and urine output data in accordance with the KDIGO criteria, some patients in the database lacked dynamic creatinine monitoring within 48 hours. This makes it impossible to accurately apply the early indicator of “an increase in creatinine of ≥0.3 mg/dL within 48 hours,” which may affect the early identification of rapidly progressive sepsis-related kidney injury. Furthermore, it limits the evaluation of the ultra-early predictive value of the ePWV for AKI. In addition, although a large sample size (n = 18,351) ensured statistical power (>99%) to detect a weak effect, with an OR of 1.05, the clinical translational value of such a small effect size requires cautious interpretation. Future studies are needed to validate the practical utility of the ePWV threshold (12.275 m/s) in guiding vascular elasticity management through prospective cohorts. Additionally, owing to the constraints of the MIMIC database, comprehensive demographic and lifestyle-related data are unavailable. This study exclusively focused on the predictive value of the baseline ePWV for AKI in patients with sepsis. Future studies should investigate the pathophysiological mechanisms by which variations in ePWV influence AKI development in the context of sepsis.

## 5. Research conclusions

This study highlights the clinical potential of ePWV in predicting AKI risk among critically ill patients with sepsis. By calculating ePWV, clinicians can achieve more precise risk stratification for AKI, gain deeper insights into individual patient risk profiles, and develop personalized treatment strategies aimed at improving patient outcomes. ePWV is a promising, straightforward, accessible, and noninvasive biomarker for predicting AKI risk in patients with sepsis. Further validation of ePWV’s predictive performance of ePWV across diverse populations is necessary to ensure the consistency and comparability of the study findings. Such efforts will facilitate broader clinical applications and integration of PWV into routine clinical practice.

Supplemental Digital Content “Table S1” is available for this article.

## Author contributions

**Conceptualization:** Yulai Wu, Zhiyi Li.

**Data curation:** Yulai Wu, Qingqing Lin, Guowei Li, Xiaoyan Liu .

**Formal analysis:** Yulai Wu, Qingqing Lin, Jing Nie.

**Investigation:** Yulai Wu, Lihua Wang, Fengmin Ge.

**Methodology:** Yulai Wu, Qingqing Lin, Zhiyi Li.

**Software:** Yulai Wu, Xiaoyan Liu.

**Supervision:** Zhiyi Li.

**Validation:** Qingqing Lin, Guowei Li, Jing Nie, Xiaoyan Liu.

**Visualization:** Yulai Wu, Xiaoyan Liu.

**Writing – original draft:** Yulai Wu.

**Writing – review & editing:** Qingqing Lin, Guowei Li, Xiaoyan Liu, Jing Nie, Lihua Wang, Fengmin Ge, Zhiyi Li.





**Figure:**
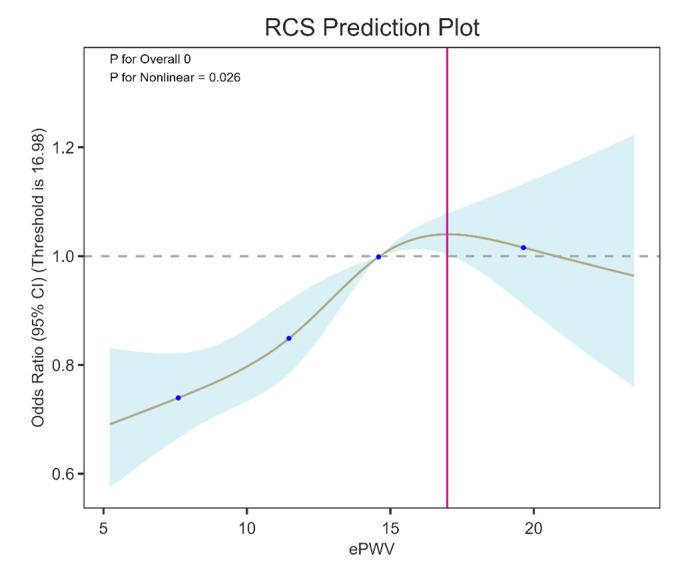


**Figure:**
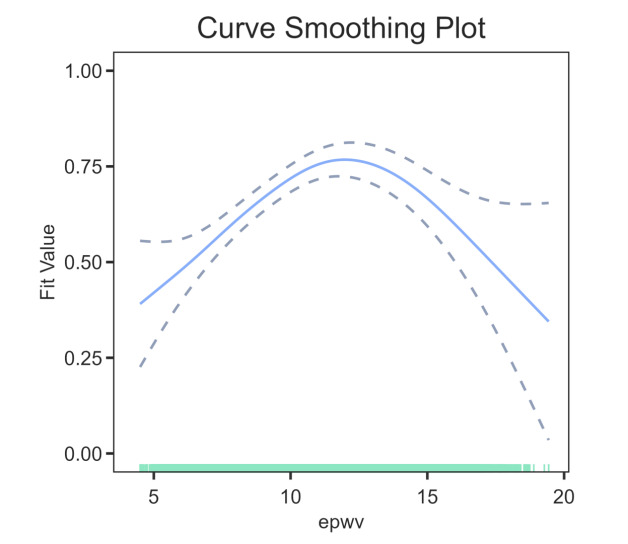


**Figure:**
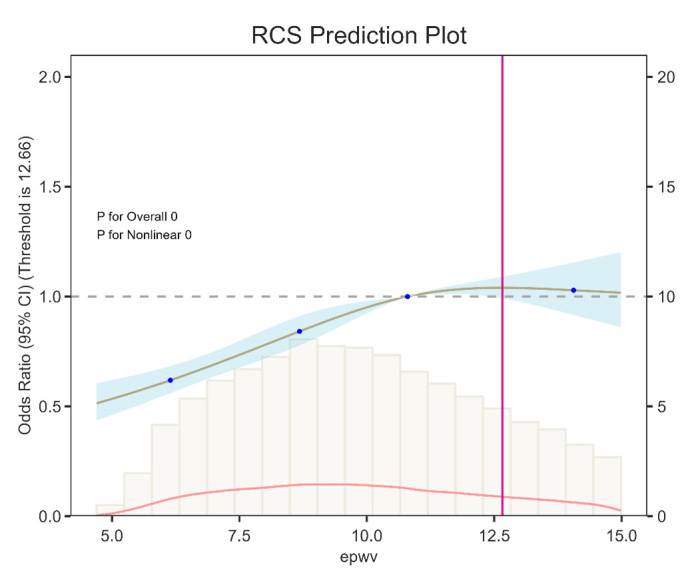


**Figure:**
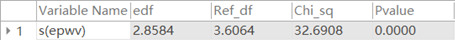


**Figure:**