# Supplementary material for: Association between estimated pulse wave velocity and acute kidney injury in critically ill sepsis patients: A MIMIC-IV database analysis
Source: Medicine (Baltimore). 2026 Jun 12;105(24):e49272. doi: 10.1097/MD.0000000000049272 (PMC13268566; doi:10.1097/MD.0000000000049272)
Supplement: Supplementary file 1 [file medi-105-e49272-s001.docx]

|  | Model 1 Model 2 Model 3 | | | | | |
| --- | --- | --- | --- | --- | --- | --- |
| Variables | OR (95%CI) | *P* | OR (95%CI) | *P* | OR (95%CI) | *P* |
| ePWV | 1.03(1.02-1.04) | <0.001 | 1.05(1.03-1.06) | <0.001 | 1.02(1.01-1.03) | 0.002 |
| ePWV |  |  |  |  |  |  |
| Q1 | Ref |  | Ref |  | Ref |  |
| Q2 | 1.16(1.05-1.28) | 0.003 | 1.16(1.05-1.28) | 0.003 | 1.07(0.96-1.21) | 0.190 |
| Q3 | 1.28(1.16-1.41) | <0.001 | 1.34(1.22-1.48) | <0.001 | 1.23(1.10-1.38) | <0.001 |
| Q4 | 1.37(1.24-1.51) | <0.001 | 1.54(1.38-1.71) | <0.001 | 1.40(1.25-1.58) | <0.004 |
| P for trend | <0.001 | | <0.001 | | <0.001 | |

**Table S2: Multi-model adjustment based on non-imputed data**

Model 1: crude model.

Model 2: adjusted race and gender.

Model3: adjusted SPO2, platelets, WBC, RBC, Albumin, Anion gap, calcium, chloride, glucose, potassium, sodium, lactate, INR, PT, SCR, HTN, CA, DM, HF, MI, CHD, COPD, LC, PNA,CVA, CB, vasopressin, and SOFA.
