# Supplementary material for: Association between estimated pulse wave velocity and acute kidney injury in critically ill sepsis patients: A MIMIC-IV database analysis
Source: Medicine (Baltimore). 2026 Jun 12;105(24):e49272. doi: 10.1097/MD.0000000000049272 (PMC13268566; doi:10.1097/MD.0000000000049272)
Supplement: Supplementary file 2 [file medi-105-e49272-s005.docx]

| Variable | Missing proportion |
| --- | --- |
| Weight | 0.74% |
| SBP | 0.90% |
| NBP | 0.90% |
| SBP | 0.88% |
| Platelet | 0.71% |
| RBC | 0.53% |
| WBC | 0.56% |
| Albumin | 29.18% |
| Anion_gap | 0.10% |
| Chloride | 0.14% |
| Glucose | 0.11% |
| Potassium | 0.20% |
| Sodium | 0.11% |
| Calcium | 27.68% |
| Lactate | 15.59% |
| INR | 4.25% |
| PT | 4.20% |
| SCR | 0.14% |
| BUN | 0.14% |

**Table: S1 Proportion of missing data**
